# Evaluation of preoperative risk factors for postpancreatectomy hemorrhage

**DOI:** 10.1007/s00423-019-01830-w

**Published:** 2019-10-24

**Authors:** Wataru Izumo, Ryota Higuchi, Takehisa Yazawa, Shuichiro Uemura, Masahiro Shiihara, Masakazu Yamamoto

**Affiliations:** Department of Surgery, Institute of Gastroenterology, Tokyo Woman’s Medical University, 8-1 Kawada-cho, Shinjuku-ku, Tokyo, 162-8666 Japan

**Keywords:** Post-pancreatectomy hemorrhage, Preoperative, Risk factor, Pancreatic fistula, Pancreatoduodenectomy

## Abstract

**Purpose:**

To investigate the risk factors for post-pancreatectomy hemorrhage (PPH).

**Methods:**

The incidence, outcome, and risk factors for PPH were evaluated in 1169 patients who underwent pancreatectomy.

**Results:**

The incidence and mortality rates of PPH were 3% and 11% in all pancreatectomies, 4% and 11% in pancreatoduodenectomy, 1% and 20% in distal pancreatectomy, and 3% and 0% in total pancreatectomy, respectively. Male sex [odds ratio (OR) 2.32], body mass index (BMI) ≥ 25 kg/m^2^ (OR 3.70), absence of diabetes mellitus (DM; HbA1c ≤ 6.2%; OR 3.62), and pancreatoduodenectomy (OR 3.06) were risk factors for PPH after all pancreatectomies. The PPH incidence was 0%, 1%, 2%, 6%, and 20% in patients with risk scores of 0 (*n* = 65), 1 (*n* = 325), 2 (*n* = 455), 3 (*n* = 299), and 4 (*n* = 25), respectively. The differences between risk-score groups 0–2 (2%) and 3–4 (7%) were significant (*P* < 0.05, OR 4.7). In patients who had undergone pancreatoduodenectomy, postoperative pancreatic fistula (POPF; OR 31.7) and absence of DM (OR 3.45) were risk factors for PPH. There was no significant association between POPF and PPH after distal pancreatectomy (*P* = 0.28). The incidence of POPF post-pancreatoduodenectomy was 20%. BMI ≥ 25 kg/m^2^ (OR 3.17), serum albumin < 3.5 g/dl (OR 1.77), absence of DM (OR 1.75), distal extrahepatic bile duct carcinoma (OR 4.05), and carcinoma of the papilla of Vater (OR 5.19) were risk factors for POPF post-pancreatoduodenectomy.

**Conclusion:**

Our study clarified the preoperative risk factors for PPH and recommends using a risk scoring system that includes “absence of DM” for predicting PPH.

**Electronic supplementary material:**

The online version of this article (10.1007/s00423-019-01830-w) contains supplementary material, which is available to authorized users.

## Introduction

Recently, the surgical technique for pancreatectomy has improved; however, pancreatectomy is associated with many complications postsurgery [1–4]. Although the frequency of postpancreatectomy hemorrhage (PPH) is not high, it results in severe adverse outcomes. The incidence rate of PPH and the mortality rate following PPH have been reported to be 3–16% and 16–36%, respectively [4–12]. The International Study Group on Pancreatic Surgery (ISGPS) defined PPH in 2007 [13]. Some studies have reported that postoperative pancreatic fistula (POPF), postoperative bile leakage, and postoperative abdominal infection are risk factors for PPH [5–12]. Although this information is useful, it includes intraoperative and postoperative factors that can only be known after surgery. Other studies reported that male sex, high body mass index (BMI), and low serum albumin (Alb) levels, which are preoperative factors, are associated with PPH [8, 10, 12, 14]. However, the preoperative risk factors for PPH have not been fully elucidated yet. Therefore, the aim of this study was to clarify the preoperative risk factors for PPH.

## Material and methods

Between 2005 and 2018, 1169 patients underwent pancreatectomy at the Department of Surgery, Institute of Gastroenterology, Tokyo Woman’s Medical University. Parameters that were evaluated included the incidence, outcomes, time of onset, location, severity, grade, and risk factors of PPH. Clinical data that were collected and examined included age; sex; preoperative BMI; white blood cell (WBC) count; serum levels of Alb, C-reactive protein (CRP), and hemoglobin A1c (HbA1c); preoperative drainage for obstructive jaundice; preoperative treatment (chemotherapy or chemoradiotherapy); operative procedure, duration of surgery; amount of intraoperative blood loss; vascular resection; pancreatojejunostomy (PJ) anastomosis method; falciform ligament wrapping of the gastroduodenal artery (GDA) stump; clipping of the GDA stump; PJ stent; pathological tissue type; pancreatic fistula; delayed gastric emptying (DGE); and postoperative bile leakage. The significant factors identified by multivariate analysis to cause PPH were defined as risk factors for PPH. The risk score was calculated, with 1 point assigned to each risk factor, and the incidence of PPH was examined for each risk score number.

The patients’ characteristics for all pancreatectomies are shown in Supplemental Table 1. Six hundred fifty-five patients (56%) were men; the median preoperative BMI level was 21.5 kg/m^2^ (range 14.3–36.8 kg/m^2^); 421 patients (36%) had diabetes mellitus (DM); 675 patients (57%) underwent pancreatoduodenectomy (PD); 211 patients (18%) had POPF grade B (15.6%, 182 patients) or C (2.5%, 29 patients); 279 (24%) patients underwent HDL wrapping of the GDA stump; none of the patients with omental grafts underwent GDA stump wrapping, and all patients underwent vascularized graft placement of the GDA stump using the falciform ligament; 26 (2%) patients underwent clipping of the GDA stump.

## Definitions

### PPH, POPF, and DGE

The PPH, POPF, and DGE grades were defined using ISGPS guidelines [13, 15, 16] and categorized into 3 (grades A, B, and C). Since the grade A cases were not clinically relevant and not a target for treatment, we only considered grade B or C cases in this study. Patients with grades B or C PPH were at risk for death, and blood transfusion, angiography, embolization, relaparotomy, and intensive treatment in the intensive care unit were needed to improve the condition of these patients.

### Diabetes mellitus

HbA1c level ≥ 6.3% was defined as DM in this study because the normal value of HbA1c in Japan is determined to be ≤ 6.2%.

### Surgical policy

Pancreatectomy with typical lymph node dissection for carcinoma involves regional lymph node dissection, and partial lymph node dissection was performed for inflammation and low malignant tumors. Regional lymph node dissection involves excision of the plexus in the right half of the SMA and dissection of the hepatoduodenal ligament from the left and right hepatic artery bifurcation to the upper margin of the pancreas. The reconstruction method was the Child’s procedure. All patients underwent PJ anastomosis for gastroenterological reconstruction, and no patient underwent pancreatogastrostomy. Usually, the GDA stump was double ligated, and beginning in 2018, additional clipping (Hem-o-lok clip®; Telefrex Medical, USA) was performed on the GDA stump. Moreover, beginning in 2010, the GDA stump was wrapped with the falciform ligament. We performed duct-to-mucosa anastomosis for PJ until 2016 and modified Blumgart anastomosis after 2017. The surgeon chose PJ stenting if the diameter of the PJ anastomosis was narrow, reconstruction was difficult, and the remaining pancreas was soft.

### Mortality rate

The rate of inpatient death related to PPH during hospitalization was used as the mortality rate in this study.

### Statistical analysis

Univariate and multivariate logistic regression analyses were performed to determine independent predictors of PPH in patients who had undergone pancreatectomy. Only factors that were significant on univariate analysis were subjected to multivariate analysis. A *P* value < 0.05 was considered statistically significant. All the analyses were performed using JMP version 12.1.9 for Windows (SAS Institute Inc., Cary, NC, USA).

## Results

### Incidence, mortality, classification, reason, and treatment of PPH after all pancreatectomies (Table 1

The incidence of PPH grades B or C was 3% in all 1169 patients undergoing pancreatectomy, 4% in the 675 patients undergoing PD, 1% in the 406 patients undergoing distal pancreatectomy (DP), and 3% in the 88 patients undergoing total pancreatectomy (TP). The incidence of late onset time, extraluminal location, high severity, bleeding from pseudoaneurysm, and interventional radiology treatment were 96%, 74%, 81%, 66%, and 63%, respectively. The median onset time of all PPH and bleeding from pseudoaneurysms from the initial surgery was 22 and 21 days, respectively. Mortality among patients with PPH was 11%. These patients died from multiple organ dysfunction caused by bleeding from pseudoaneurysms. The overall mortality rate within 30 and 90 days was 0.3% (3/1169) and 0.6% (7/1169), respectively. Mortality from PPH within 30 and 90 days was 0.2% (2/1169) and 0.3% (3/1169), respectively.

**Table 1:** Incidence, mortality, classification, reason, and treatment of post-pancreatectomy hemorrhage grades B or C after all pancreatectomy

Category	Definition	All	PD	DP	TP
Total number of PPHs		*n* = 35	*n* = 27	*n* = 5	*n* = 3
Incidence of PPH		3% (35/1169)	4% (27/675)	1% (5/406)	3% (3/88)
Mortality among patients with PPH		4 (11%)	3 (11%)	1 (20%)	0 (0%)
Median onset time from initial surgery (days), (range)		22 (0–65)	24 (0–47)	9 (0–27)	2 (0–65)
Time of onset	Early/late	4 (4%)/31 (96%)	1 (4%)/26 (96%)	1 (20%)/4 (80%)	2 (67%)/1 (33%)
Location	Intraluminal/extraluminal	9 (26%)/26 (74%)	7 (26%)/20 (74%)	1 (20%)/4 (80%)	1 (33%)/2 (67%)
Severity	Mild/severe	9 (19%)/26 (81%)	5 (19%)/22 (81%)	2 (40%)/3 (60%)	2 (67%)/1 (33%)
Reason	Pseudoaneurysm GDA/HA/SMA/SPA Bleeding from the stump of the pancreas, greater omentum or retroperitoneal	22 (66%) 13/5/1/3 7 (19%)	18 (66%) 12/5/1/0 5 (19%)	3 (60%) 1/0/0/2 1 (20%)	1 (33%) 0/0/0/1 1 (33%)
	Gastrointestinal bleeding	6 (15%)	4 (15%)	1 (20%)	1 (33%)
Treatment	Interventional radiology Conservative treatment with blood transfusion Endoscopic treatment Surgery Interventional radiology plus surgery	19 (63%) 6 (15%) 5 (11%) 4 (7%) 1 (4%)	17 (63%) 4 (15%) 3 (11%) 2 (7%) 1 (4%)	2 (40%) 1 (20%) 1 (20%) 1 (20%) 0 (0%)	0 (0%) 1 (33%) 1 (33%) 1 (0%) 0 (0%)

*PPH* postpancreatectomy hemorrhage, *GDA* gastroduodenal artery, *HA* hepatic artery, *SMA* superior mesenteric artery, *SPA* splenic artery, *PD* pancreatoduodenectomy, *DP* distal pancreatectomy, *TP* total pancreatectomy

Classification categories were defined by the International Study Group of Pancreatic Surgery [7]

Time of onset: early hemorrhage means that bleeding occurred ≤ 24 h after the end of the operation; late hemorrhage means that bleeding occurred > 24 h after the end of the index operation

Location: intraluminal hemorrhage means bleeding from the gastrointestinal tract; extraluminal hemorrhage means bleeding from the abdominal cavity

Severity: Mild means that the patient’s condition was not severe enough to necessitate invasive treatment; severe means that the patient’s condition was poor, and it was necessary to perform intensive treatment

### Univariate and multivariate analyses of risk factors for PPH after all pancreatectomies (Table 2) and PPH rate by independent risk factors (Supplemental Table 2)

In multivariate analysis, male sex (odds ratio [OR] 2.32), BMI ≥ 25 kg/m^2^ (OR 3.70), absence of DM (OR 3.62), and PD (OR 3.06) were significant independent risk factors for PPH in all patients undergoing pancreatectomy (*P* < 0.05). When assessing using these risk factors, the incidence of PPH after all pancreatectomies in patients with a risk score of 0 (*n* = 65), 1 (*n* = 325), 2 (*n* = 455), 3 (*n* = 299), and 4 (*n* = 25) was 0%, 1%, 2%, 6%, and 20% (*P* < 0.0001), respectively (Supplemental Table 2). The differences between risk-score groups 0–2 (2%) and 3–4 (7%) were significant (*P* < 0.05, OR 4.7).

**Table 2:** Univariate and multivariate analyses of risk factors for post-pancreatectomy hemorrhage grades B or C after all pancreatectomy

			Univariate			Multivariate	
Risk factors	Definition	*n*	PPH	OR (95% CI)	*P* value	OR (95% CI)	*P* value
Age (years)	< 65 ≥ 65	493 676	12 (2.4%) 23 (3.4%)	1 1.41 (0.70–2.87)	0.34		
Sex	Female Male	518 651	8 (1.5%) 27 (4.2%)	1 2.76 (1.24–6.12)	0.0013	1 2.32 (1.02–5.26)	0.034
BMI (kg/m^2^)	< 25 ≥ 25	1011 158	24 (2.4%) 11 (7.0%)	1 3.08 (1.48–6.41)	0.0027	1 3.70 (1.71–8.04)	0.0009
WBC (/ul)	< 8000 ≥ 8000	1093 76	3 (4.0%) 32 (2.9%)	1 1.36 (0.41–4.56)	0.62		
Alb (g/dl)	≥ 3.5 < 3.5	1040 129	26 (2.5%) 9 (7.0%)	1 2.92 (1.34–6.39)	0.0071	1 2.17 (0.95–4.98)	0.066
CRP (mg/dl)	< 1 ≥ 1	1055 114	29 (2.8%) 6 (5.3%)	1 1.97 (0.80–4.84)	0.14		
DM	Presence Absence	421 748	6 (1.4%) 29 (3.9%)	1 2.79 (1.15–6.78)	0.023	1 3.62 (1.46–8.99)	0.0056
Pretreatment *	With Without	37 1132	1 (2.7%) 34 (3.0%)	1 1.11 (0.15–8.37)	0.92		
Operative procedure	DP	406	5 (1.2%)	1		1	
	PD TP	675 88	27 (4.0%) 3 (3.4%)	3.34 (1.28–8.75) 2.83 (0.66–12.1)	0.014 0.16	3.06 (1.12–8.36) 3.27 (0.73–14.7)	0.029 0.12
Operation time (min)	< 360 ≥ 360	597 572	15 (2.5%) 20 (3.5%)	1 1.41 (0.71–2.77)	0.33		
Blood loss (ml)	< 1200 ≥ 1200	1010 159	26 (2.6%) 9 (5.7%)	1 2.27 (1.04–4.94)	0.039	1 1.50 (0.66–3.43)	0.34
Vascular resection	Without	967	29 (3.0%)	1	0.98		
	With	202	6 (3.0%)	0.99 (0.41–2.42)			

*Pretreatment was preoperative chemotherapy or chemoradiotherapy

**Delayed gastric emptying was defined by the International Study Group on Pancreatic Surgery

*PPH* postpancreatectomy hemorrhage, *OR* odds ratio, *CI* confidence interval, *BMI* body mass index, *WBC* white blood cells, *Alb* albumin, *CRP* C-reactive protein, *DM* diabetes mellitus, *DP* distal pancreatectomy, *PD* pancreatoduodenectomy, *TP* total pancreatectomy, *DGE* delayed gastric emptying

### Univariate and multivariate analyses of risk factors for PPH after PD (Table 3)

Because PD was risk factor of PPH after all pancreatectomies, we evaluated risk factors for PPH after PD, excluding DP and TP. In multivariate analysis, POPF (OR 31.7) and absence of DM (OR 3.45) were independent risk factors for PPH after PD (*P* < 0.05). There was no significant difference in the incidence of PPH between patients with and without falciform ligament wrapping of the GDA stump [5.7% (11/276) and 2.8% (16/399), respectively; *P* = 0.059]. The incidence of POPF after PD was 20%.

**Table 3:** Univariate and multivariate analyses of risk factors for postpancreatectomy hemorrhage grades B or C after pancreatoduodenectomy

			Univariate			Multivariate	
Risk factors	Definition	*n*	PPH	OR (95% CI)	*P* value	OR (95% CI)	*P* value
Period	2005–2011 2012–2018	324 351	9 (2.8%) 18 (5.1%)	1 1.89 (0.84–4.27)	0.12		
Age (years)	< 65 ≥ 65	251 424	8 (3.2%) 19 (4.5%)	1 1.43 (0.61–3.30)	0.41		
Sex	Female Male	264 411	7 (2.7%) 20 (4.9%)	1 1.88 (0.78–4.51)	0.15		
BMI (kg/m^2^)	< 25 ≥ 25	595 80	20 (3.4%) 7 (8.8%)	1 2.76 (1.13–6.74)	0.021	1 2.22 (0.77–6.43)	0.14
WBC (/ul)	< 8000 ≥ 8000	628 47	25 (4.0%) 2 (4.3%)	1 1.07 (0.25–4.67)	0.93		
Alb (g/dl)	≥ 3.5 < 3.5	563 112	17 (3.0%) 10 (8.9%)	1 2.68 (1.17–6.12)	0.016	1 2.04 (0.79–5.28)	0.14
CRP (mg/dl)	< 1 ≥ 1	577 98	22 (3.8%) 5 (5.1%)	1 1.36 (0.50–3.67)	0.56		
DM	Presence Absence	241 434	4 (1.7%) 23 (5.3%)	1 3.32 (1.13–9.70)	0.021	1 3.45 (1.06–11.3)	0.040
Pre-drainage	Without With	404 271	13 (3.2%) 14 (5.2%)	1 1.64 (0.76–3.54)	0.21		
Pretreatment *	With Without	29 646	1 (3.5%) 26 (4.0%)	1 1.17 ‘0.15–8.97)	0.87		
Operation time (min)	< 360 ≥ 360	220 455	9 (4.1%) 18 (4.0%)	1.04 (0.46–2.34) 1	0.93		
Blood loss (ml)	< 1200 ≥ 1200	566 109	18 (3.2%) 9 (8.4%)	1 2.81 (1.23–6.43)	0.011	1 1.74 (0.65–4.66)	0.27
Vascular resection	Without With	566 109	22 (3.9%) 5 (4.6%)	1 1.19 (0.44–3.21)	0.73		
PJ anastomosis method	Duct-to mucosa Modified Blumgart	580 95	23 (4.0%) 4 (4.2%)	1 1.06 (0.36–3.15)	0.91		
Falciform ligament wrapping of GDA stump	Without With	399 276	16 (2.8%) 11 (5.7%)	1 2.13 (0.97–4.66)	0.059		
Clipping of GDA stump	Without With	657 18	26 (4.0%) 1 (5.6%)	1 1.43 (0.18–11.1)	0.75		
PJ stent	Without With	318 357	8 (2.5%) 19 (5.3%)	1			
				2.18 (0.94–5.05)	0.059		
Pathological tissue type	PDAC IPMN DEBDC PVC Others	261 139 149 75 50	6 (2.3%) 3 (2.2%) 12 (8.1%) 4 (5.3%) 2 (4.0%)	1		1	
				0.94 (0.23–3.82) 3.74 (1.37–10.2) 2.40 (0.66–8.75) 1.78 (0.35–9.07)	0.93 0.010 0.18 0.49	1.13 (0.22–5.72) 1.49 (0.47–4.75) 0.81 (0.19–3.49) 2.14 (0.34–13.3)	0.88 0.50 0.78 0.42
POPF**	None or A B or C	542 133	3 (0.6%) 24 (18.1%)	1 39.6 (11.8–133.7)	< 0.0001	1 31.7 (8.94–112.2)	< 0.0001
DGE***	None or A B or C	626 49	25 (4.0%) 2 (4.1%)	1 1.02 (0.23–4.45)	0.98		
Bile leakage	Without With	665 10	26 (3.9%) 1 (10.0%)	1 2.73 (0.33–22.4)	0.33		

*Pretreatment was preoperative chemotherapy or chemoradiotherapy

**Postoperative pancreatic fistula was defined by the International Study Group on Pancreatic Surgery

***Postoperative delayed gastric emptying was defined by the International Study Group on Pancreatic Surgery

*PPH* postpancreatectomy hemorrhage, *OR* odds ratio, *CI* confidence interval, *BMI* body mass index, *WBC* white blood cell, *Alb* albumin, *CRP* C-reactive protein, *DM* diabetes mellitus, *GDA* gastroduodenal artery, *PJ stent* pancreatojejunostomy stent, *PDAC* pancreatic ductal adenocarcinoma, *DEBDC* distal extrahepatic bile duct carcinoma, *IPMN* intraductal papillary mucinous neoplasm, *PVC* carcinoma of the papilla of Vater, *POPF* postoperative pancreatic fistula, *DGE* delayed gastric emptying

### Univariate and multivariate analyses of risk factors for POPF after PD (Table 4)

Because POPF was risk factor for PPH after PD, we assessed risk factors for POPF after PD. In multivariate analysis, BMI ≥ 25 kg/m^2^ (OR 3.17), Alb < 3.5 g/dl (OR 1.77), absence of DM (OR 1.75), distal extrahepatic bile duct carcinoma (OR 4.05), and carcinoma of the papilla of Vater (PVC) (OR 5.19) were independent risk factors for POPF after PD.

**Table 4:** Univariate and multivariate analyses of preoperative risk factors for postoperative pancreatic fistula grades B or C after pancreatoduodenectomy

			Univariate			Multivariate	
Risk factors	Definition	*n*	POPF	OR (95% CI)	*P* value	OR (95% CI)	*P* value
Age (years)	< 65 ≥ 65	251 424	42 (16.8%) 91 (21.5%)	1 1.36 (0.91–2.04)	0.14		
Gender	Female Male	264 411	37 (14.0%) 96 (23.4%)	1 1.87 (1.23–2.83)	0.0024	1 1.57 (0.99–2.46)	0.05002
BMI (kg/m^2^)	< 25 ≥ 25	595 80	106 (17.8%) 27 (33.8%)	1 2.35 (1.41–3.91)	0.0015	1 3.17 (1.81–5.56)	< 0.0001
WBC (/ul)	< 8000 ≥ 8000	628 47	120 (19.1%) 13 (27.7%)	1 1.62 (0.83–3.16)	0.16		
Alb (g/dl)	≥ 3.5 < 3.5	563 112	99 (17.6%) 34 (30.4%)	1 1.93 (1.22–3.06)	0.0050	1 1.77 (1.07–2.93)	0.027
CRP (mg/dl)	< 1 ≥ 1	577 98	108 (18.7%) 25 (25.5%)	1 1.49 (0.90–2.45)	0.12		
DM	Presence Absence	241 434	35 (14.5%) 98 (22.6%)	1 1.72 (1.12–2.62)	0.0123	1 1.75 (1.10–2.78)	0.019
Pre-drainage	Without With	404 271	65 (16.1%) 68 (25.1%)	1 1.75 (1.19–2.56)	0.0042	1 0.69 (0.40–1.17)	0.17
Pretreatment *	With Without	646 29	126 (19.5%) 7 (24.1%)	1 1.31 (0.55–3.14)	0.54		
Pathological tissue type	PDAC IPMN DEBDC PVC Others	262 139 149 75 50	30 (11.5%) 18 (13.0%) 49 (32.9%) 29 (38.7%) 7 (14.0%)	1		1	
				1.15 (0.62–2.15) 3.79 (2.27–6.32) 4.88 (2.67–8.89) 1.26 (0.52–3.05)	0.66 < 0.0001 < 0.0001 0.61	1.14 (0.60–2.20) 4.05 (2.15–7.62) 5.19 (2.70–9.97) 1.05 (0.42–2.65)	0.69 < 0.0001 < 0.0001 0.92

*Pretreatment was preoperative chemotherapy or chemoradiotherapy

*POPF* postoperative pancreatic fistula, *OR* odds ratio, *CI* confidence interval, *BMI* body mass index, *WBC* white blood cell, A*lb* albumin, *CRP* c-reactive protein, *DM* diabetes mellitus, *PJ stent* pancreatojejunostomy stent, *PDAC* pancreatic ductal adenocarcinoma, *DEBDC* distal extrahepatic bile duct carcinoma, *IPMN* intraductal papillary mucinous neoplasm, *PVC* carcinoma of the papilla of Vater

### Association between POPF and PPH after DP and PD

The incidence of POPF after DP was 19% (78/406). There was no significant difference in the incidence of POPF between DP and PD [20% (133/675), *P* = 0.84]. Unlike with PD where the OR of 31.7, there was also no significant association between POPF and PPH after DP [3% (2/78) in patients with POPF and 1% (3/328) in patients without POPF; *P* = 0.28].

## Discussion

This study indicates that absence of DM (HbA1c level ≤ 6.2%) is an independent risk factor for PPH after all pancreatectomies and PD and for POPF after PD; a risk scoring system including the absence of DM may be useful for predicting PPH before surgery. By determining the value of HbA1c level (≤ 6.2%), we can predict PPH more easily. This is a new finding that has not been reported before.

In recent years, PPH has a low frequency, but it has a poor outcome. POPF, DGE, wound infection, and abdominal abscess are major complications of PD [3, 4, 17]. Previous literatures reported that the incidence of PPH was only 3–16%; however, mortality among patients with PPH was 16–36% [4–12]. In this study, the incidence and mortality rates of patients with PPH were 3% (35/1169) and 11% (4/35), respectively (Table 1), after all pancreatectomies. PPH was the major cause of death within 30 days after pancreatectomy. Previous literatures also reported that short-term outcomes after pancreatectomy were better in high-volume centers than in low-volume centers (mortality rate 0.9–6.0% vs. 13.0–18.8%) [9, 18–20].

Some previous reports about intraoperative and postoperative factors showed that vascular resection, pancreaticogastrostomy, postoperative bile leakage, postoperative abdominal infection, and especially POPF were independent risk factors for PPH [5–12]. PPH from a pseudoaneurysm of the GDA stump usually results from POPF [9, 21], and the median onset time of PPH was reported as 5–13 days (range 0–58 days) [6, 10, 12]. In our study, similar results were noted, and it is necessary to pay attention to the onset time from initial surgery (median onset time 22 days, range 0–65 days; Table 1). Previous literatures have mentioned preoperative risk factors for PPH such as male sex, high BMI, and low Alb level [8, 10, 12, 14]; our study also showed a similar result. Additionally, in our study, “absence of DM (HbA1c level ≤ 6.2%)” was one of the significant independent risk factors for PPH after all pancreatectomies and PD, which has not been reported.

Insulin acts to promote secretion of pancreatic exocrine cells [22]. It has been reported that the pancreatic exocrine function declined as insulin secretion decreased and the exocrine pancreatic glands atrophied [23]. This means that in patients with DM, the pancreatic parenchyma atrophies and becomes hard, and in patients without DM, the pancreatic parenchyma is thick and soft. Traditionally, patients with a soft pancreas, thick parenchyma, and thin pancreatic duct are considered at risk for POPF after PD [24–27]. Pancreatic exocrine function is an important determinant of POPF after PD [28]. In pancreatic carcinoma, it has been reported that pancreatic parenchyma atrophy is seen from the onset of illness and the main pancreatic duct is dilated [29]. In the same way, in a patient with intraductal papillary mucinous neoplasm, the main pancreatic duct is slightly dilated and the pancreatic parenchyma is relatively decreased and atrophic. In contrast, in patients with distal extrahepatic bile duct carcinoma, main pancreatic duct dilation and pancreatic parenchyma atrophy are rare. Patients with a thick and soft pancreatic parenchyma have a high possibility of POPF and a high risk of PPH after pancreatectomy. In situations where there is no clear definition of the thickness and hardness of the pancreas and it is difficult to judge them preoperatively, the presence or absence of DM (HbA1c level ≤ 6.2%) is considered to be one of the good indicators.

Previous literatures reported that omental flaps or grafts around various anastomoses after PD could reduce the incidence of POPF and PPH [30, 31]. In this study, the statistical analysis did not show a significant benefit of wrapping the GDA stump for preventing PPH. We believe we cannot draw sufficient statistical conclusions because of differences in surgical techniques and instruments, differences in applications and methods of round ligament wrapping, and the limited use of clips in recent cases. Hence, further clinical evaluation for this is necessary. In addition, pancreatic surgery in patients at high risk for developing PPH may require new precautions to further reduce the incidence of PPH.

### Limitations

Patients from different periods over the 14-year span of the study underwent different diagnostic and treatment modalities owing to the advances in techniques that occurred over time; these variations may have skewed the outcomes of patients treated during the different periods of the study. Moreover, our investigation had a retrospective design and was performed at a single institution; the biases inherent in such settings cannot be completely excluded.

## Conclusion

Male sex, BMI ≥ 25 kg/m^2^, absence of DM (HbA1c level ≤ 6.2%), and PD are independent risk factors for PPH after all pancreatectomies. A risk scoring system including the new preoperative risk factor “absence of DM” would be useful for predicting PPH.

## Electronic supplementary material

ESM 1 (DOCX 31 kb)
