# Supplementary material for: Evaluation of preoperative risk factors for postpancreatectomy hemorrhage
Source: Langenbecks Arch Surg. 2019 Oct 24;404(8):967–74. doi: 10.1007/s00423-019-01830-w (PMC6935390; doi:10.1007/s00423-019-01830-w)
Supplement: Supplementary file 1 — (DOCX 31 kb) [file 423_2019_1830_MOESM1_ESM.docx]

Supplemental Table 1. Patients’ characteristics for all pancreatectomies

| Category | Overall (n=1169) |
| --- | --- |
| Median age (years, range) | 66 (15-87) |
| Sex  Male  Female | 651 (56%)  518 (44%) |
| Median BMI (kg/m^2^, range) | 21.5 (14.3-36.8) |
| Median WBC (/ul, range) | 5100 (2080-15920) |
| Median Alb (g/dl, range) | 4.1 (2.2-5.2) |
| Median CRP (mg/dl, range) | 0.1 (0.01-18.1) |
| Median HbA1c (%, range) | 6.0 (4.3-14.4) |
| DM  Presence  Absence | 421 (36%)  748 (64%) |
| Preoperative treatment *  With  Without | 37 (3%)  1132 (97%) |
| Preoperative drainage for obstructive jaundice  With  Without | 344 (29%)  825 (71%) |
| Surgical procedure  PD  DP  TP | 675 (57%)  406 (35%)  88 (8%) |
| Operation time (min, range) | 355 (97-952) |
| Blood loss (ml, range) | 514 (5-18000) |
| Vascular resection  With  Without | 202 (17%)  967 (83%) |
| PJ stent  With  Without | 412 (35%)  757 (65%) |
| Pathological tissue type  PDAC  IPMN  DEBDC  PVC  Others | 467 (40%)  270 (23%)  149 (13%)  75 (6%)  208 (18%) |
| POPF **  None or A  B or C | 958 (82%)  211 (18%) |
| DGE ***  None or A  B or C | 1101 (94%)  68 (6%) |
| Postoperative bile leakage  None or A  B or C | 1158 (99%)  11 (1%) |

Abbreviations: BMI: body mass index, WBC: white blood cell, Alb: albumin, CRP: C-reactive protein, HbA1c: hemoglobin A1c, DM: diabetes mellitus, PD: pancreaticoduodenectomy, DP: distal pancreatectomy, TP: total pancreatectomy, PJ stent: pancreatojejunostomy stent, PDAC: pancreatic ductal adenocarcinoma, IPMN: intraductal papillary mucinous neoplasm, DEBDC: distal extrahepatic bile duct carcinoma, PVC: carcinoma of the papilla of Vater, POPF: postoperative pancreatic fistula, DGE: delayed gastric emptying

* Pretreatment was preoperative chemotherapy or chemoradiotherapy.

** Postoperative pancreatic fistula was defined by the International Study Group on Pancreatic Surgery.

*** Postoperative delayed gastric emptying was defined by the International Study Group on Pancreatic Surgery.

Supplemental Table 2. Incidence of post-pancreatectomy hemorrhage grade B or C after all pancreatectomies

****kokom

***kokom

**

*

| Risk score | PPH grade B or C |
| --- | --- |
| 0 (n=65)  1 (n=325)  2 (n=455)  3 (n=299)  4 (n=25) | 0 (0%)  3 (1%)  10 (2%)  17 (6%)  5 (20%) |

*P=0.29, **P=0.16, ***P=0.013, ****P=0.021

Footnote

Using multivariate analysis, the risk factors for PPH grade B or C after all pancreatectomies were male sex, BMI ≥25 kg/m^2^, absence of DM, and PD. The risk score was calculated, with 1 point assigned to each risk factor, and the incidence of PPH was examined for each risk score number.

Abbreviations: PPH: post-pancreatectomy hemorrhage, BMI: body mass index, DM: diabetes mellitus, PD: pancreatoduodenectomy
